# Emergence of Asynchronous Local Clocks in Excitable Media

**DOI:** 10.1371/journal.pone.0142490

**Published:** 2015-11-11

**Authors:** Richard Carl Gerum, Ben Fabry, Claus Metzner

**Affiliations:** Department of Physics, University of Erlangen-Nuremberg, Erlangen, Germany; Universidad Rey Juan Carlos, SPAIN

## Abstract

Excitable media such as the myocardium or the brain consist of arrays of coupled excitable elements, in which the local excitation of a single element can propagate to its neighbors in the form of a non-linear autowave. Since each element has to pass through a refractory period immediately after excitation, the frequency of autowaves is self-limiting. In this work, we consider the case where each element is spontaneously excited at a fixed average rate and thereby initiates a new autowave. Although these spontaneous self-excitation events are modelled as independent Poisson point processes with exponentially distributed waiting times, the travelling autowaves lead collectively to a non-exponential, unimodal waiting time distribution for the individual elements. With increasing system size, a global ‘clock’ period *T* emerges as the most probable waiting time for each element, which fluctuates around *T* with an increasingly small but non-zero variance. This apparent synchronization between asynchronous, temporally uncorrelated point processes differs from synchronization effects between perfect oscillators interacting in a phase-aligning manner. Finally, we demonstrate that asynchronous local clocks also emerge in non-homogeneous systems in which the rates of self-excitation are different for all individuals, suggesting that this novel mechanism can occur in a wide range of excitable media.

## Introduction

Self-organized oscillations are ubiquitous in complex systems that consist of distributed excitable elements with non-linear interactions. A well-known example are the Belousov-Zhabotinsky reactions [1, 2], in which cyclic color changes and other spatio-temporal patterns emerge from simple chemical reaction processes. A typical phenomenon in this type of systems is the so-called autowave—a wave-like propagation of excitation between the individual elements that does not obey the linear superposition principle. In this context, the ‘excitation’ of an element can be defined as just one particular state of the element, out of a discrete set of possible states. Due to this generality, autowaves are found in a variety of fields, including neural systems [3, 4], in the spreading of diseases [5–7], in forest fires [8–10], or in the wave dynamics of penguin huddles [11, 12].

A simple way to describe the dynamics of such systems is the use of cellular automaton (CA) models, such as the Greenberg-Hasting model [13]. While the dynamics of this system and its stable spatio-temporal patterns have been investigated extensively [14–16], the dynamics of repeatedly and randomly triggered Greenberg-Hasting automata remained unexplored. The repeated triggering of autowaves in active media is important, e.g. for the electrical excitation in cardiac tissue where irregular autowaves can lead to ventricular fibrillation and sudden cardiac arrest [17] or arrhythmia [18]. Here, we extend the Greenberg-Hasting model by the possibility of spontaneous excitation of any cell and investigate the implications for the global statistics of autowaves. We show that for large system sizes, the distributions of time intervals between autowave triggering events turn from an exponential to a unimodal distribution with a clearly defined maximum, representing the timescale of a global clock.

## Model

A cellular automaton (CA) is a regular array of *N* cells, each of which can be in a number of discrete states. The states can change during each time step according to fixed rules that take into account the present state of the cell and its neighbors. [19]

We first consider the simplest case of a one-dimensional CA with three states (0,1,2) that couple only to the nearest neighbors. According to the Greenberg-Hasting model, every cell starts in the resting state 0 and turns to the excited state 1 when a neighbor is in the excited state. In the next time step, the excited state turns to the refractory state 2 during which it cannot be excited again. The refractory state changes back to the resting state in the following time step. We extend this model by introducing the possible spontaneous activation of a cell (spontaneous triggering) in the resting state with a probability *q*.

- 0 ⇒ 1 if neighbour is 1, or with probability *q*
- 1 ⇒ 2 always
- 2 ⇒ 0 always

**Fig 1:**
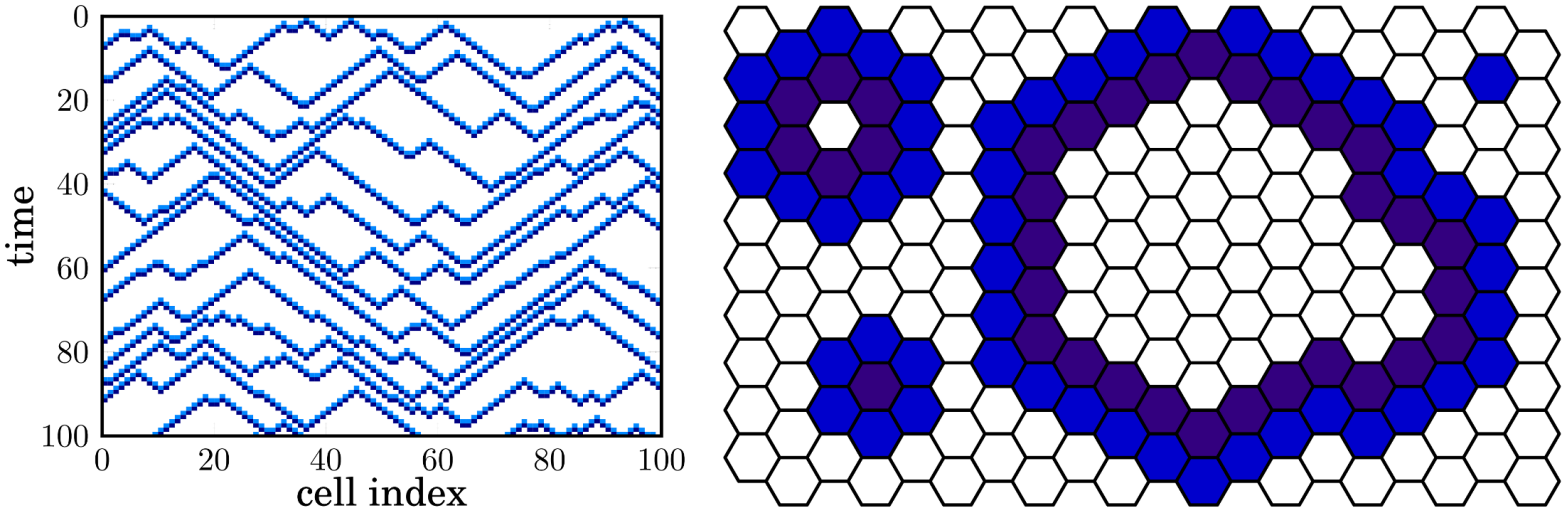
Illustration of 1D and 2D cellular automaton models for repeatedly triggered autowaves. The 1D system consists of a linear row (x-direction) of *N* = 100 cells. States are decoded by color (0: white, 1: light blue, 2: dark blue). Each line corresponds to a global wave event. The 2D system consists of a hexagonal lattice. With a probability of *q* = 0.02, a resting cell is spontaneously excited (state 0 → state 1). This corresponds to the tips of triangular shapes in the space-time diagram of the 1D system. Two autowaves merge and annihilate when the wavefronts touch, resulting in an inverse tip or valley.

The dynamics of this model can be visualized in a space-time diagram (Fig 1). Here, autowaves appear as triangular shapes with the triggering cell at the top. The front of an autowave spreads from the triggering cell to either side at a constant speed (of one cell per time step), retaining a constant width (of two cells). Several autowaves can propagate simultaneously in the system. Due to the constant propagation speed, an autowave cannot overtake another from behind, although two opposing wavefronts may collide. In this case, the two wavefronts merge at the collision point, contributing to the zig-zag-shaped excitation patterns of each global wave event (Fig 1).

Most applications of the model require two spatial dimensions. Since quadratic two-dimensional grids can produce artifacts due to the small number of possible wave propagation directions [20], we use here a hexagonal grid with six neighbors for each cell (see Fig 1). The update rules for the cell states are the same as in the 1D model.

## Effective triggering rate

First, we investigate the influence of the effective triggering rate *R*_eff_ of the cells, defined as the average number of time steps each cell spends in the activated state relative to the total number of simulation steps. Due to the propagation of activation to neighboring cells, *R*_eff_ is always larger than the spontaneous triggering rate *R*_spon_ = 1/*T*_spon_, which is approximately (*R*_spon_ would be exactly proportional to *q* if the time spent in states 1 and 2 are neglected) proportional to the triggering probability *q*. With a larger number *N* of cells, *R*_eff_ is expected to increase.

**Fig 2:**
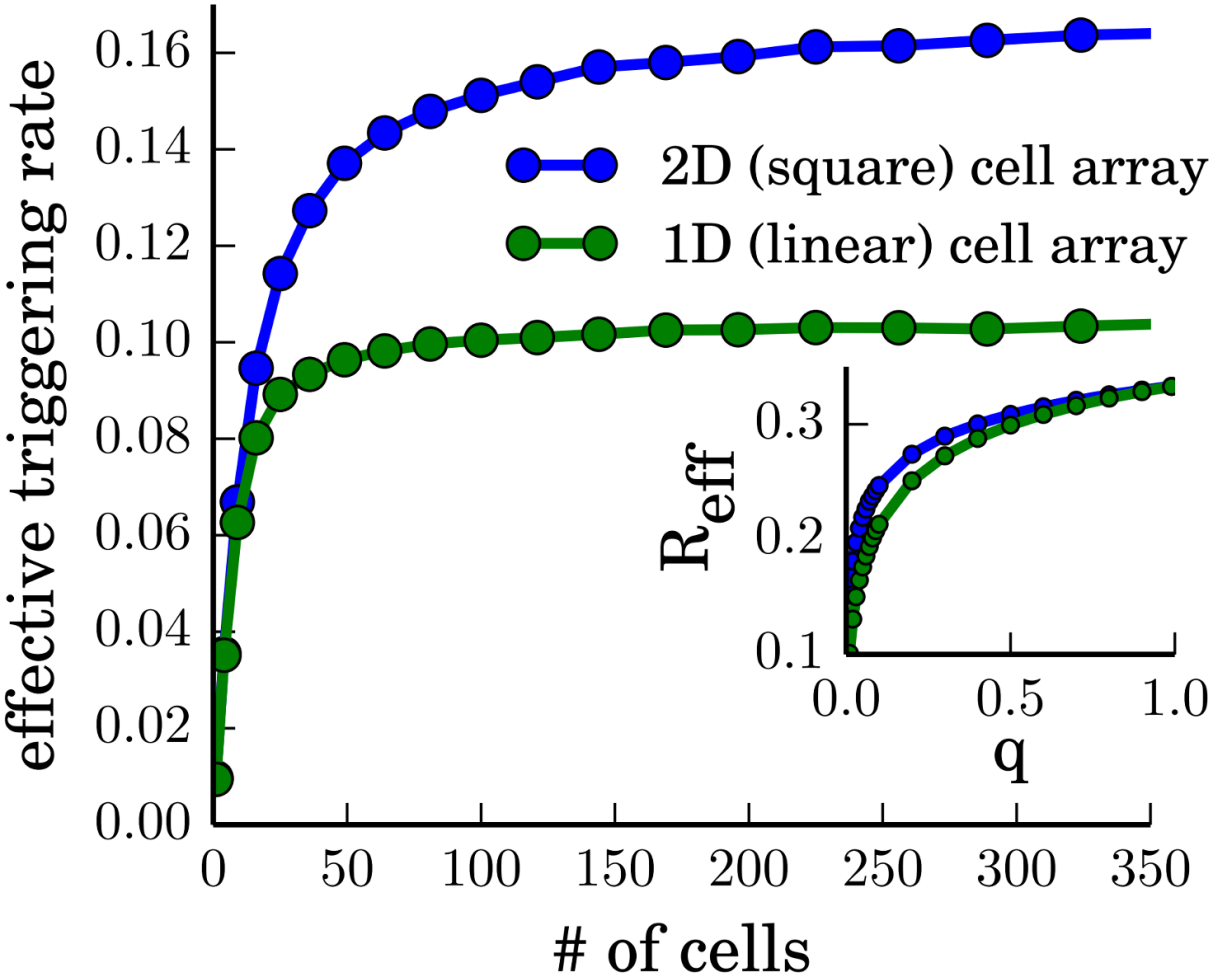
Dependence of the effective triggering rate *R*_eff_ on the system size *N* for a fixed spontaneous triggering probability *q* = 0.01. 1D corresponds to a 1 × *N* array, 2D corresponds to a $\sqrt{N}\times \sqrt{N}$ array of hexagonal cells. The inset shows the dependence of *R*_eff_ on *q* for a fixed number of cells *N* = 100.

Numerical simulations confirm these expectations (Fig 2) but show an asymptotic saturation of *R*_eff_ for large cell numbers *N*. The asymptotic value depends on *q* and the geometry of the system.

Without autowaves, *R*_eff_ would only be determined by the spontaneous triggering events and would therefore increase linearly with *q*. At *q* = 1, the system would reach the upper limit of the effective triggering rate, which is 1/3 because the cells have to cycle through the states 2 and 0 before the next excitation. With autowaves, each spontaneous triggering event can lead to the additional excitation of a certain number of neighboring cells, until the wavefront is annihilated by collision with another wavefront. Therefore, *R*_eff_ is always larger or equal to *q*/3. However, due to the mutual annihilation of autowaves, the average number of neighbors which can be triggered by a given autowave is decreasing with the density of simultaneous autowaves in the system. For this reason, *R*_eff_ first increases rapidly with *q*, but for larger *q* the slope decreases until *R*_eff_ reaches the upper limit, 1/3, at *q* = 1 (Fig 2 inset).

For relatively small systems, geometric effects also influence the effective triggering rate: for a linear configuration, a wave takes a longer time *T*_spread_ to pass through the whole system. If a wave starts at one end, a spontaneous triggering event at the other end can lead to a separate wave only after the first wavefront has already passed. Otherwise, the two wavefronts merge and annihilate, resulting in only one global wave event (Fig 1). Therefore, as the number of cells *N* in the row increases, there are more spontaneous triggering events, but this is counteracted by the increasing wave spreading time *T*_spread_, explaining the observed saturation behavior. For a square cell array of size $\sqrt{N}\times \sqrt{N}$, the spreading time *T*_spread_ is smaller. Therefore, *R*_eff_ saturates for larger *N* and approaches higher values. (Fig 2). This geometric dependence vanishes for *q* → 1, where more and more cells are triggered spontaneously and not by the interaction with their neighbors (Fig 2 inset).

## Triggering interval distribution

**Fig 3:**
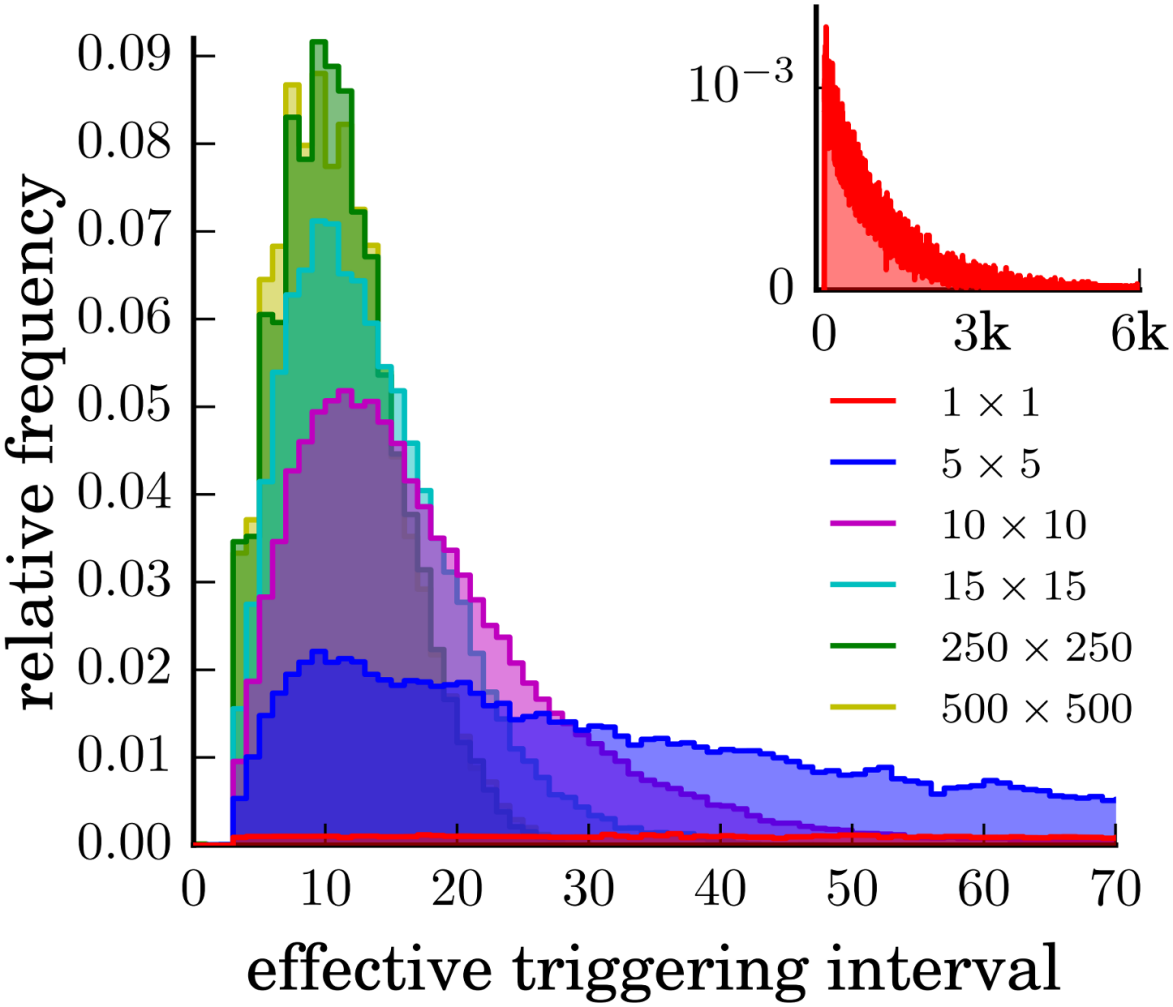
Distribution of waiting times between triggering events. For a system with *q* = 0.001 and no interactions (inset), the waiting times are distributed according to an exponential distribution. For square arrays of hexagonal cells with *N* = 5 × 5, *N* = 10 × 10, *N* = 15 × 15, *N* = 250 × 250 and *N* = 500 × 500 interacting cells, waiting time distributions have an asymmetric peak at a non-zero value of *T*_eff_. The width of the peak decreases with the size *N* of the system.

A more detailed description of the triggering statistics is provided by the distribution *p*(*T*_eff_) of effective triggering intervals between two successive activated states of the same cell. For a single, isolated cell, this distribution is exponential (Apart from a small deviation as intervals of length 0 or 1 cannot occur), since spontaneous triggering was modeled as a Poisson point process (Fig 3 inset). For multiple interacting cells, the triggering statistics of each cell is more complex.

With increasing numbers of interacting cells, *p*(*T*_eff_) evolves continuously from an exponential (gamma distribution with parameter *k* = 1) to a gamma distribution (with *k* ≠ 1) (Fig 3). The latter distribution has a well-defined mode; the most probable triggering interval changes from zero to a finite value *T*_peak_. As the size *N* of the system grows, the peak position moves to smaller values, corresponding to an increasing effective triggering rate. Simultaneously, the width of the distribution around *T*_peak_ decreases. This means that collectively, the interacting cells form an increasingly accurate clock. Although each wave can be triggered by a different cell, every cell is triggered in roughly regular intervals. In the limit of very large *N*, *p*(*T*_eff_) converges to a limiting distribution which still has a finite variance due to the stochastic nature of the system.

## Systems with internal clocks

The Poisson point process is the simplest model for spontaneous triggering. However, many biological systems already possess internal clocks so that even without interactions, the distribution of the triggering intervals has a maximum at a non-zero value. Here we model the spontaneous triggering intervals *T*_spon_ as a gamma distribution *p*(*T*_spon_) with average triggering interval *μ* = *k* ⋅ *θ* and standard deviation $\sigma =\sqrt{k}\cdot \theta$.

$$\begin{array}{c} p\left(x=T_{\text{spon}}\right)=\frac{x^{k-1}e^{-\frac{x}{\theta }}}{\theta^{k}\Gamma \left(k\right)} \end{array}$$ (1)

**Fig 4:**
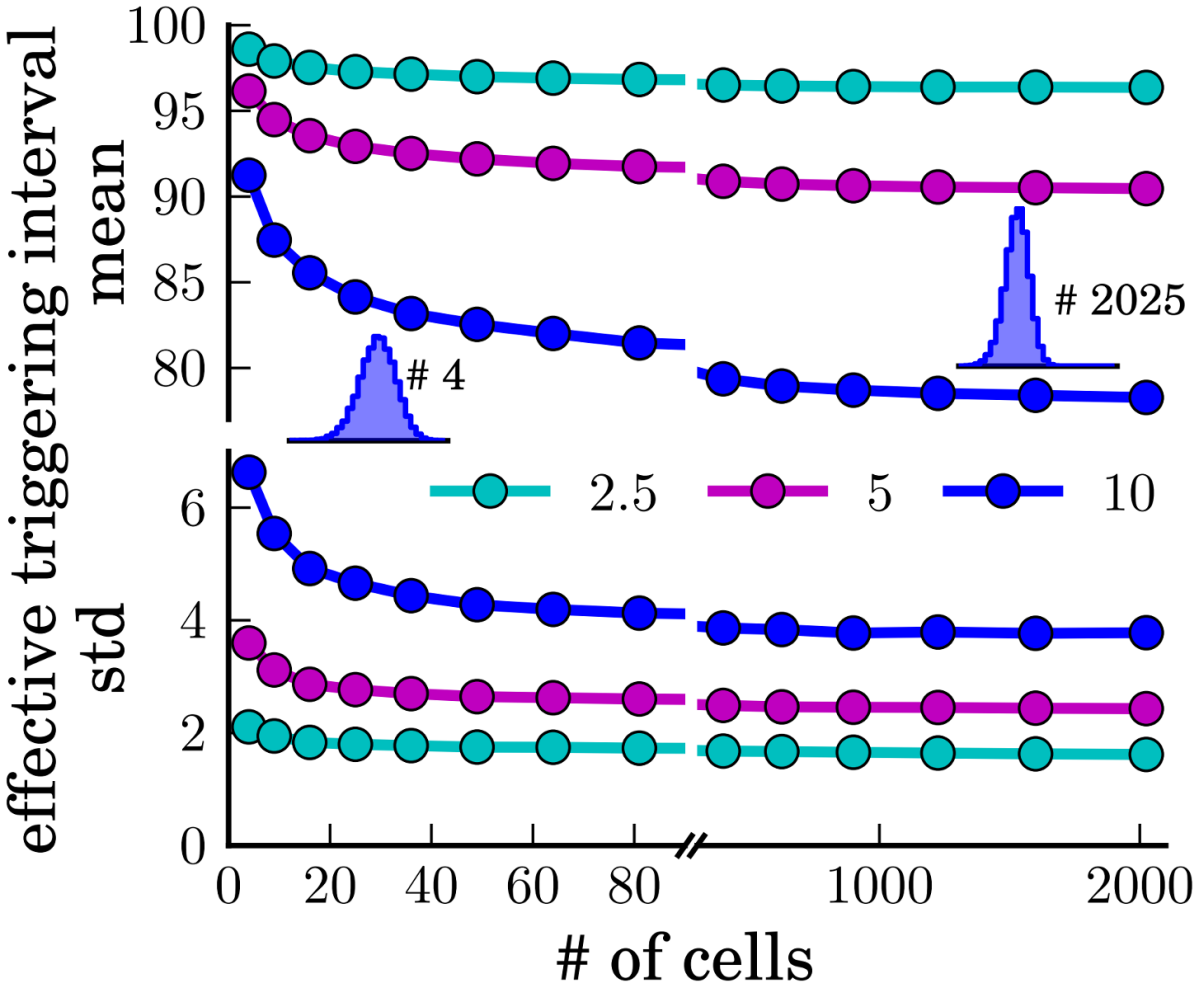
Mean (top) and standard deviation (bottom) of the distributions *p*(*T*_eff_) of the effective triggering intervals, for gamma distributed spontaneous triggering distributions *p*(*T*_spon_) with a mean of 100 and standard deviations of 10 (blue), 5 (magenta) and 2.5 (cyan). For a large number of cells *N*, the mean and the standard deviation of *p*(*T*_eff_) both saturate at a value below the mean and standard deviation of *p*(*T*_spon_).

To investigate how the interactions between autowaves interfere with the internal clocks of the cells, we consider square arrays of hexagonal cells, a mean spontaneous triggering interval of *μ* = 100 and three different standard deviations *σ* = 10, 5 and 2.5. We then compute the mean and standard deviation of the resulting distribution of effective triggering intervals for different system sizes (Fig 4).

We find that the mean triggering interval and its standard deviation decreases with increasing system size and saturates at a value that depends on *μ* and *σ*. Hence, similar to our findings in systems without internal clock, the interaction of autowaves reduces the fluctuations of the triggering intervals.

## Wave model of linear medium

**Fig 5:**
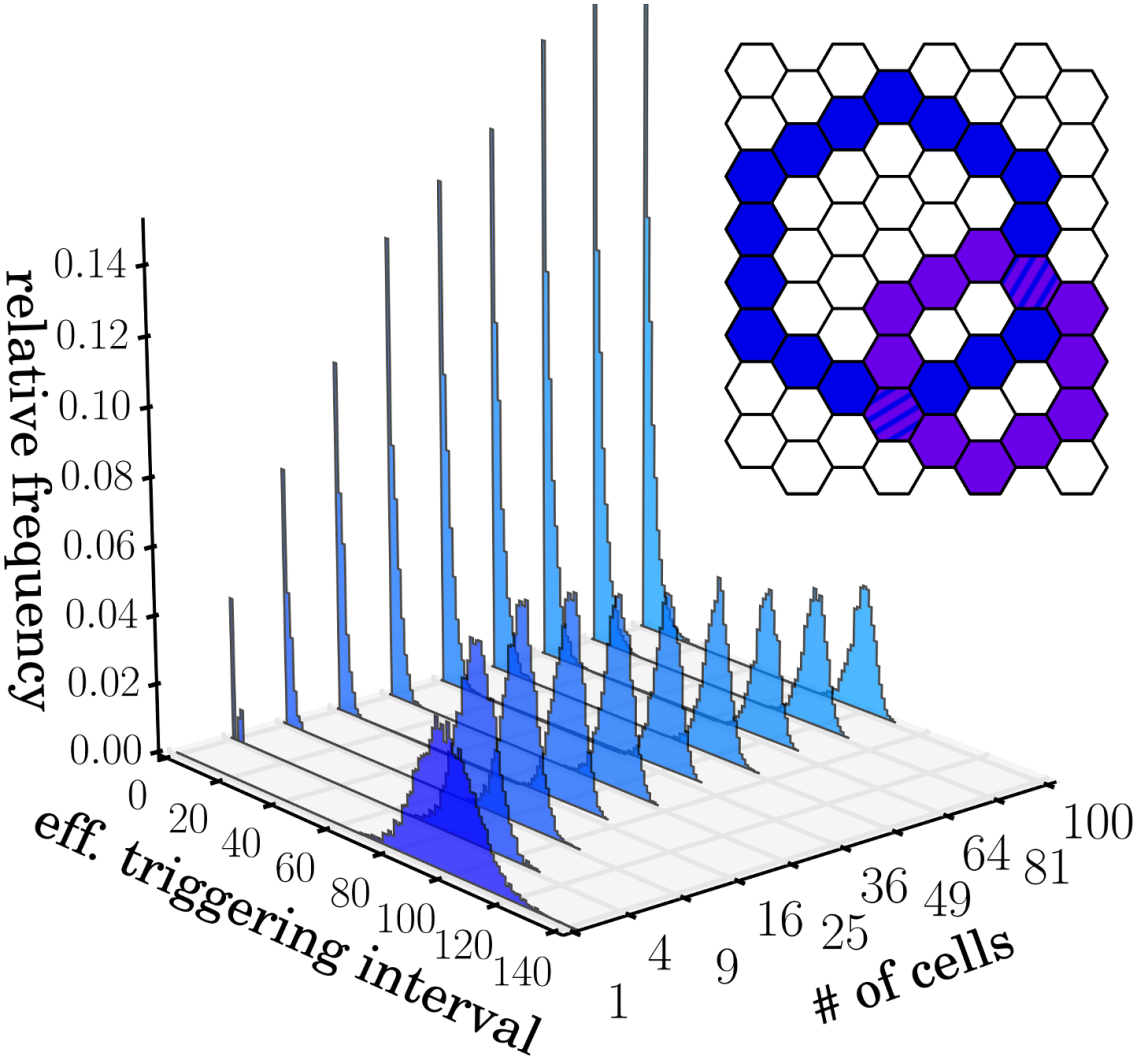
Distribution of effective triggering intervals in the linear medium model. There are two separate peaks visible in the histogram: one centered around 80, corresponding to that of the Greenberg-Hasting model, and one around zero. The inset shows two wavefronts passing through each other.

To demonstrate that the non-linear interactions of the autowaves are responsible for the emergence of a global clock, we investigate a model without wave front annihilations. In this model, waves are still triggered spontaneously, transfer the excitation to the neighbors, and thus propagate through the system. However, colliding waves now pass through each other freely (Fig 5 inset).

To implement this system, we extended the CA model in a way that resembles lattice gas models [21], yet without collision rules. This required us to remove the refractory state and to represent the information about the direction of wave movement in another way: the state of every cell encodes a superposition of wavefronts moving in any of the 6 possible directions. (Each cell can contain one or more “arrows” in 6 possible directions given by the hexagonal lattice. At every timestep, each arrow moves to the adjacent cell to which it has pointed. A cell is treated as “exited” (corresponding to state 1 in the Greenberg-Hasting model) when it contains at least one arrow.)

Simulations with the linear medium model reveal a more complex distribution of triggering intervals (Fig 5) than in the active medium model: the triggering intervals still have a peak at a non-zero value smaller than *T*_spon_, which becomes even smaller as the system size grows. However, a second, exponential peak appears at a triggering interval of zero, because each cell is eventually triggered by every wave that runs through the system. For larger system sizes, this second peak increasingly dominates the distribution. Therefore, the interactions in the linear wave model disturb the internal clock of the cells and decrease the accuracy of the clock.

## Summary

In this article, we explored the effect of spontaneous random triggering of individual cells in excitable media. We showed that for large systems, the coupling by autowaves leads to the emergence of an accurate clock. This effect occurs regardless of the underlying spontaneous triggering process of the individual cells. In particular, we have demonstrated the effect for the two cases of a Poisson point process (corresponding to cells without an internal clock) and a gamma distributed process (for cells with an internal clock). We also showed that the clock requires non-linear, merging autowaves. The effect does not emerge in a linear system where the superposition principle holds.

It is different from systems where a each agent already has a prefect individual clock and where these clocks start to synchronize in phase and frequency as soon as the coupling strength exceeds a critical value (Kuramoto phase transition [22]). The case described in this work, where the agents agree on a global time scale without synchronization of their phases, may be relevant for numerous biological systems such as for the electrical excitation in the heart [23] or in the brain [3, 24] and may also play a role in the collective motion of densely packed individuals such as humans in a crowd, or the huddling dynamics of emperor penguins [12]. In this context, an important feature of our model is that even without a phase synchronization or the emergence of spatially stable patterns, the individual clocks agree on a common frequency.

Our simple model can be extended in various ways. For instance, the coupling need not be restricted to nearest neighbors (see S1 Appendix). The average spontaneous triggering intervals may vary amongst the individual cells (see S2 Appendix), and the activated and refractory states may have longer lifetime (see S3 Appendix), similar to cardiomyocytes or neurons. Finally, the fundamental lattice structure need not be quadratic, hexagonal, or even regular, but instead could contain complex interaction network topologies, such as scale free networks. With these extensions, we expect that our model is applicable to a wide range of systems, such as the spreading of human diseases by global traffic, or the spreading of opinions in social networks.

## Supporting Information

S1 Appendix Effect of coupling range. Simulation of a system with increased coupling range.
(PDF)

S2 Appendix Effect of heterogenous cell populations. Simulation of a heterogenous system with cells of different values of *μ*_*i*_.
(PDF)

S3 Appendix Effect of longer refractory periods. Simulation of a system with a longer refractory period then one time step.
(PDF)
