# Supplementary material for: Emergence of Asynchronous Local Clocks in Excitable Media
Source: PLoS One. 2015 Nov 11;10(11):e0142490. doi: 10.1371/journal.pone.0142490 (PMC4641646; doi:10.1371/journal.pone.0142490)
Supplement: S1 Appendix — Simulation of a system with increased coupling range. (PDF) [file pone.0142490.s001.pdf]

S1 Appendix. Effect of coupling range

Above, we have investigated systems with nearest neighbor interactions (n_6). For larger coupling ranges n (Fig. S1), all of the main results hold qualitatively, however, the saturation value of the mean effective triggering intervals decreases. The standard deviation σ of $T_{\rm eff}$ decreases with n even stronger than the mean, indicating that a larger coupling range enhances the accuracy of the global clock.

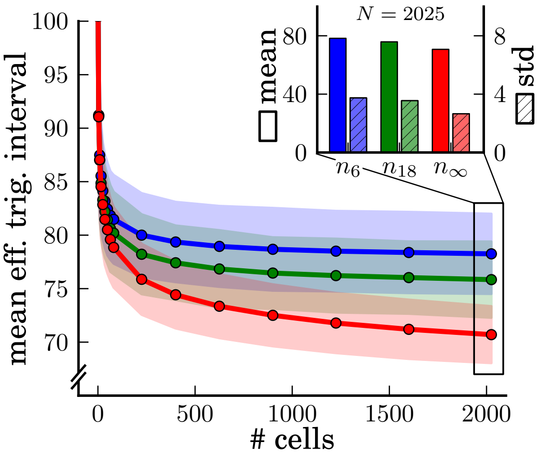

Figure S1. Mean effective triggering interval as a function of system size for different coupling ranges (n_6 corresponds to six direct neighbours, n_{18} to neighbours within range two and n_∞ to a global coupling of all cells). With increasing coupling range, the saturation value decreases. The inset shows mean values and standard deviations of $p(T_{\rm eff})$ for a system size $N = 2025$. With increasing coupling range, the standard deviation decreases.
