# Supplementary material for: Emergence of Asynchronous Local Clocks in Excitable Media
Source: PLoS One. 2015 Nov 11;10(11):e0142490. doi: 10.1371/journal.pone.0142490 (PMC4641646; doi:10.1371/journal.pone.0142490)
Supplement: S2 Appendix — Simulation of a heterogenous system with cells of different values of μ i. (PDF) [file pone.0142490.s002.pdf]

S2 Appendix. Effect of heterogenous cell populations

In the simulations above, the average triggering interval μ was constant for all cells in the system. However, most biological system consist of heterogenous populations, where not all individuals can be described by the same parameters. To investigate the effect of heterogenity on the clock formation, we assigned different values μ_i for each cell, using a uniform distribution within the range from $\mu_{\min}$ to $\mu_{\max}$.

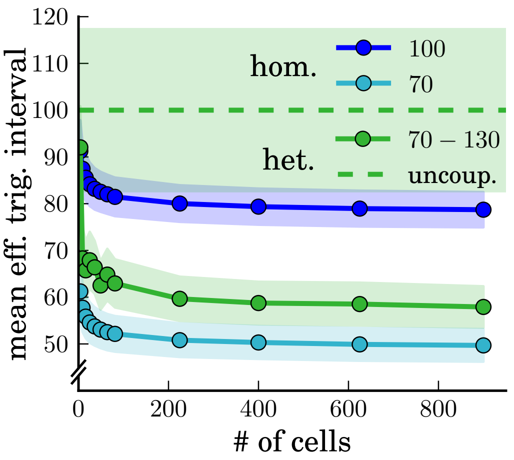

Figure S1. Comparison of mean (lines) and standard deviation (shaded areas) of effective triggering interval for a homogeneous and a heterogeneous system. The blue line corresponds to a homogeneous system with $\mu = 100, \sigma = 10$, the solid green line corresponds to a system where every cell has a different μ, uniformly distributed between 70 and 130. The light blue line corresponds to a homogeneous system with $\mu = 70$. Green dashed line and green shaded area indicate the expected mean triggering interval and standard deviation for a heterogeneous system without coupling (uniform distribution of μ between 70 and 130).

We find that the mean effective triggering interval of a heterogeneous population also saturates for high N (Fig. S1) but with a lower value than in the homogeneous case (for the same average μ of the spontaneous triggering interval distribution). Interestingly, the asymptotic mean T_{eff} does not coincide with the minimum μ of the distribution, implying that the system is not dominated by the fastest members of the population. Nevertheless, the impact of the members with $\mu = \mu_{\min}$ is larger than that of those with $\mu = \mu_{\max}$. It is also remarkable that the fluctuation σ of the effective triggering interval in the coupled heterogeneous system is significantly smaller than the distribution of μ amongst the individuals.
