# Supplementary material for: Emergence of Asynchronous Local Clocks in Excitable Media
Source: PLoS One. 2015 Nov 11;10(11):e0142490. doi: 10.1371/journal.pone.0142490 (PMC4641646; doi:10.1371/journal.pone.0142490)
Supplement: S3 Appendix — Simulation of a system with a longer refractory period then one time step. (PDF) [file pone.0142490.s003.pdf]

S3 Appendix. Effect of longer refractory periods

In the simulation, we always used a refractory period of 1 time step, which means that a cell in the refractory period changed its state back to the exitable state within one time step. Many real systems have different time scales for the spreading of activation and the recovery from the activation. To implement longer refractory periods, we required each cell to pass through a sequence of n_{ref} refractory states before going back to the excitable state.

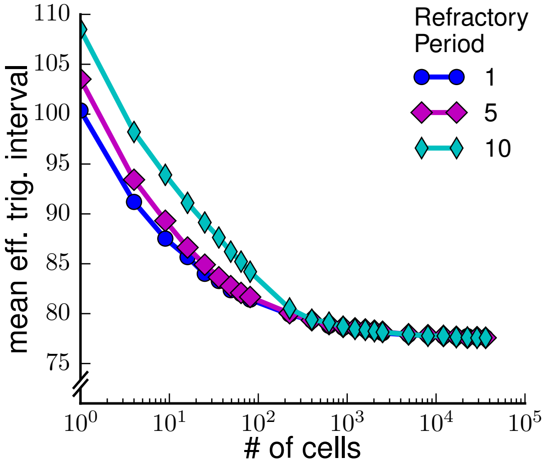

Figure S1. Comparison of mean of effective triggering interval for systems with $\mu = 100, \sigma = 10$ and a refractory period of 1, 5 and 10. The mean effective triggering interval was higher for longer refractory periods and smaller systems, but the effects of different refractory periods vanishes for larger systems.

The simulations reveal (Fig. S1), that the mean effective triggering interval and therefore the emergence of asynchronous local clocks, is not qualitatively affected by the refractory period.
